# Outpatient Visit Current Procedural Terminology Code Level Selection Trends in Hand Surgery Following Criteria Changes by the American Medical Association

**DOI:** 10.7759/cureus.27125

**Published:** 2022-07-21

**Authors:** Jack G Graham, Kyle Plusch, Michael Rivlin, Samir Sodha, Greg G Gallant, Pedro Beredjiklian

**Affiliations:** 1 Division of Hand Surgery, Rothman Orthopaedic Institute, Philadelphia, USA; 2 Department of Orthopaedic Surgery, Hackensack University Medical Center, New York, USA

**Keywords:** medical decision-making, hand surgery, coding, evaluation and management, current procedural terminology

## Abstract

Introduction: Beginning on January 1, 2021, the American Medical Association (AMA) and the Centers for Medicare and Medicaid Services (CMS) implemented considerable revisions with regard to the outpatient evaluation and management (E/M) criteria dictating the Current Procedural Terminology (CPT) code level selection. The primary goal of the current study was to determine how the recent E/M coding criteria changes have impacted code level selection by orthopedic hand surgeons in the outpatient setting.

Materials and methods: All outpatient visits within the hand and wrist surgery division of a single orthopedic practice were collected during two timeframes: March 1, 2019, to June 30, 2019, and March 1, 2021, to June 30, 2021. Procedure codes and insurance categories were collected for each visit. The primary endpoint analyzed was the visit level of care based on CPT E/M codes. For each timeframe, we determined the number of total visits that were coded at each level and expressed them as a percentage of the total visits for that time period. The insurance plan billed for each visit was recorded and classified as Medicare, Medicaid, Workers' Compensation, or commercial.

Results: In 2019, prior to the billing level requirement changes, 7.2% of all visits were billed as level 2, 84.8% of all visits were billed as level 3, and 7.8% of all visits were billed as level 4. In 2021, 1.9% of visits were billed as level 2, 47.3% of visits were billed as level 3, and 50.5% of visits were billed as level 4. Level 1 and 5 visits did not exceed 0.5% in either timeframe. Within each insurance category, the proportion of visit levels of care followed a similar trend of reduced level 2 and 3 visits and increased level 4 visits from 2019 to 2021.

Conclusion: We noted a significant trend toward higher code level selection following the recent code level changes, and we anticipate these recent code selection trends to have major financial implications moving forward.

## Introduction

On January 1, 2021, the Centers for Medicare and Medicaid Services (CMS) implemented considerable revisions to the outpatient evaluation and management (E/M) criteria dictating Current Procedural Terminology (CPT) code level selection [1,2]. It has been well-documented that physicians spend a disproportionate amount of time working on the electronic health record (EHR), often at the expense of face-to-face time with the patient [3,4]. As a part of their “Patients over Paperwork” initiative, the CMS sought to help diminish administrative burden and simplify the documentation required of physicians to justify code level selection in the outpatient setting [1,5,6]. These revisions represented the first major overhaul in E/M coding in over two decades.

These recent changes place the onus of the coding level on the complexity of medical decision-making (MDM) and not on the documentation requirements on the history and physical examination sections of the medical record as had been the case under the previous system [2]. Physicians now have the flexibility to document the pertinent history and physical examination findings in the EHR “as medically appropriate” to support their MDM. While the CPT codes have remained the same, the level of service (LOS) is now determined by MDM or total time spent by the physician on the date of the encounter. Time spent includes reviewing pertinent data or notes, face-to-face interaction, and time spent documenting or placing orders in the EHR on the day of the encounter only. MDM takes into account the number and complexity of problems addressed, the amount and/or complexity of data reviewed and analyzed, and the risk of complications and/or morbidity/mortality associated with the management of the patient’s conditions [1]. Under the previous system, the extensive documentation required to reach a higher LOS may have deterred subspecialists from higher-level code selection. These changes are particularly impactful in fields such as hand surgery, where the appropriate history and physical examination can often be especially focused.

The purpose of the current study was to determine how the recent E/M coding criteria changes have impacted code level selection by orthopedic hand surgeons in the outpatient setting. We hypothesized that the new emphasis on MDM would be associated with higher-level CPT code selection by hand surgeons. Given that visit complexity is directly tied to reimbursement, the secondary outcome measured was the number of corresponding relative value units (RVUs) per visit in this same set of patients under the new coding criteria.

## Materials and methods

Following Institutional Review Board approval, including a waiver of informed consent per institutional protocol, we performed a billing database search to identify all in-person outpatient visits among 18 fellowship-trained hand surgeons within a single orthopedic practice during two timeframes: March 1, 2019, to June 30, 2019, and March 1, 2021, to June 30, 2021. While the billing level change occurred on January 1, 2021, the year 2020 was not included due to the significant disruption of in-person office visits associated with the coronavirus disease 2019 (COVID-19) pandemic. Patient demographics and procedure codes were collected for each visit, and internal billing records were reviewed to collect the insurance category and plan billed for each visit. Using the Physician Fee Schedule available through the CMS website, corresponding RVUs from 2019 to 2021 were also collected [7].

The primary endpoint analyzed was the visit level of care based on CPT E/M codes. Historically, many outpatient clinic visits have been billed for using one of 10 five-digit CPT codes, which represent both patient status (new versus established) and visit complexity based on LOS. All new patient visits have been represented by a CPT E/M code 9920_, with the final digit ranging from 1 (low complexity) to 5 (high complexity). Established patient visit E/M codes begin with 9921_, with the final digit also ranging from 1 (low complexity) to 5 (high complexity). The specific criteria for each E/M code selection are described in Table 1 below [1]. Work RVUs are assigned to each of these E/M CPT codes by the CMS as outlined in their Physician Fee Schedule [7]. Consults, post-operative visits, and fracture care follow-up (within the global period) have been unaffected by the recent changes by the American Medical Association (AMA) and CMS. Thus, we omitted these patient visits from our analysis.

**Table 1:** Medical decision-making criteria in evaluation and management code selection Table adapted from the 2021 American Medical Association guidelines regarding CPT E/M code changes [1]. LOS = level of service; E/M = evaluation and management; CPT = Current Procedural Terminology; MDM = medical decision-making.

LOS	E/M CPT code(s)	Time (minute) (alternative)	MDM complexity	Possible problem combinations	Data			Risk
					Category 1: Tests and documents	Category 2: Interpretation	Category 3: Management/discussion	
1	99211 (est)	N/A	Medically appropriate	No requirement	No requirement			N/A
2	99202 (new) 99212 (est)	15-29, 10-19	Straightforward	1 self-limited	No requirement			Minimal
3	99203 (new) 99213 (est)	30-44, 20-29	Low	1 acute, uncomplicated; 1 chronic, stable; 2 self-limited	Combo of 2: ・External note review ・Test result review ・Ordering test	・Assessment utilizing independent historian	No requirement	Low
					Requirement: 1 of 2 categories			
4	99204 (new) 99214 (est)	45-59, 30-39	Moderate	1 acute injury, complicated; 1 acute illness with systemic symptoms; 1 new problem, undiagnosed; 1 chronic, progression, or exacerbation; 2 chronic, stable	Combo of 3: ・External note review ・Test result review ・Ordering test ・Assessment utilizing independent historian	・Independent interpretation of tests performed by another healthcare professional	・Discussion with outside healthcare professional	Moderate
					Requirement: 1 of 3 categories			
5	99205 (new) 99215 (est)	60-74, 40-54	High	1 acute injury/illness, threatening bodily function and/or life; 1 chronic, severe progression, or exacerbation	Combo of 3: ・External note review ・Test result review ・Ordering test ・Assessment utilizing independent historian	・Independent interpretation of tests performed by another healthcare professional	・Discussion with outside healthcare professional	High
					Requirement: 2 of 3 categories			

Starting January 1, 2021, the code 99201 was removed due to historic underutilization, meaning it is no longer possible to code a new patient visit as level 1. For each time period, we determined the number of total visits that were coded at each level and expressed them as a percentage of the total visits for that time period. The insurance plan billed for each visit was recorded and classified as Medicare, Medicaid, Workers' Compensation, or commercial. All categorical variables were compared with chi-square analysis, and continuous variables were compared with a two-sample t-test.

## Results

Over the eight months of data collection, there were 34,593 total visits among 26,935 unique patients (Table 2). From March 1, 2019, to June 30, 2019 period, there were 15,904 outpatient visits; the corresponding period in 2021 had 18,689 visits.

**Table 2:** Proportion of visit levels by year Data are presented as the number of visits (% of that year’s total visits). LOS = level of service; E/M = evaluation and management; CPT = Current Procedural Terminology.

Visit type	LOS	E/M CPT code(s)	Year		P-value
			2019	2021	
All visits	1	99201, 99211	10 (0.1)	4 (0.02)	0.056
	2	99202, 99212	1143 (7.2)	353 (1.9)	<0.001
	3	99203, 99213	13492 (84.8)	8834 (47.3)	<0.001
	4	99204, 99214	1236 (7.8)	9437 (50.5)	<0.001
	5	99205, 99215	23 (0.1)	61 (0.3)	<0.001>
New patient	1	99201	2 (0.03)	0 (0.00)	0.1
	2	99202	49 (0.8)	14 (0.2)	<0.001
	3	99203	5683 (93.0)	3913 (47.8)	<0.001
	4	99204	360 (5.9)	4211 (51.5)	<0.001
	5	99205	14 (0.2)	41 (0.5)	0.009
Established patient	1	99211	8 (0.1)	4 (0.04)	0.2
	2	99212	1094 (11.2)	339 (3.2)	<0.001
	3	99213	7809 (79.7)	4921 (46.8)	<0.001
	4	99214	876 (8.9)	5226 (49.7)	<0.001
	5	99215	9 (0.1)	20 (0.2)	0.06

The proportion of visit billing levels changed substantially from 2019 to 2021. Prior to the billing level requirement changes, the majority of visits were billed as level 3 (84.8%), compared to an almost even split of level 3 and level 4 visits after changes (47.3% level 3; 50.5% level 4). The difference in the number of visits billed at levels 2, 3, and 4 between the two timeframes was significant (p < 0.001). These data are represented in Table 2, which also breaks down the levels of care for new patient visits and established patient visits separately.

Insurance data were available for 33,360 (96.4%) of the patient visits and are depicted in Table 3. Commercial insurance providers were billed for 64.9% of visits, 24.9% of visits were billed to Medicare, 9.0% of visits were billed to Workers’ Compensation, and 1.2% of visits were billed to Medicaid. Within each insurance category, the proportion of visit levels of care followed a similar trend of significantly reduced level 2 and level 3 visits and greatly increased level 4 visits from 2019 to 2021. Per the CMS Physician Fee Schedule, mean RVUs billed per visit increased significantly (p < 0.001) for all visits, new patient visits, established patient visits, and each insurance category (Table 4) [7].

**Table 3:** Proportion of visit levels by insurance type and year Data are presented as the number of visits (% of that year’s total visits). LOS = level of service; E/M = evaluation and management; CPT = Current Procedural Terminology.

Insurance type	LOS	E/M CPT codes	Year		P-value
			2019	2021	
Commercial	1	99201, 99211	4 (0.04)	2 (0.02)	0.24
	2	99202, 99212	679 (7.3)	216 (1.7)	<0.001
	3	99203, 99213	8005 (86.5)	6039 (48.8)	<0.001
	4	99204, 99214	562 (6.1)	6101 (49.3)	<0.001
	5	99205, 99215	6 (0.1)	24 (0.2)	0.012
Medicare	1	99201, 99211	2 (0.1)	0 (0.0)	0.14
	2	99202, 99212	266 (6.7)	72 (1.7)	<0.001
	3	99203, 99213	3400 (86.2)	1797 (41.2)	<0.001
	4	99204, 99214	272 (6.9)	2475 (56.8)	<0.001
	5	99205, 99215	4 (0.1)	13 (0.3)	0.047
Medicaid	1	99201, 99211	0 (0.0)	0 (0.0)	N/A
	2	99202, 99212	16 (7.3)	5 (2.6)	0.027
	3	99203, 99213	183 (83.6)	111 (56.6)	<0.001
	4	99204, 99214	20 (9.13)	76 (38.8)	<0.001
	5	99205, 99215	0 (0.0)	4 (2.0)	0.034
Workers’ Compensation	1	99201, 99211	2 (0.1)	1 (0.1)	0.88
	2	99202, 99212	158 (8.4)	40 (3.6)	<0.001
	3	99203, 99213	1360 (72.3)	489 (43.4)	<0.001
	4	99204, 99214	349 (18.6)	580 (51.5)	<0.001
	5	99205, 99215	11 (0.6)	16 (1.4)	0.019

**Table 4:** Average relative value units (RVUs) billed per visit

	2019	2021	Percent increase	P-value
All visits	1.161	1.824	57.1%	<0.001
New patient visits	1.479	2.123	43.5%	<0.001
Established patient visits	0.963	1.591	65.2%	<0.001
Commercial insurance	1.133	1.811	59.8%	<0.001
Medicare	1.181	1.877	59.0%	<0.001
Medicaid	1.163	1.734	49.1%	<0.001
Workers’ Compensation	1.219	1.823	49.5%	<0.001

## Discussion

The CPT code system dates back to 1966, one year after Congress created Medicare under the Social Security Act [8]. The AMA has overseen consistent revisions of the system ever since. In the year 2000, the CPT system was officially named the coding standard for all United States health care [8]. Today, each CPT code is five digits long and corresponds to nearly any healthcare service that can be billed for [5,8]. These codes are subcategorized into one of the following groups: medicine, surgery, radiology, anesthesia, E/M, pathology, and laboratory. E/M codes are the predominant subcategory utilized in the outpatient setting, including the hand surgery clinic.

Prior to recent changes by the AMA and CMS, the level of complexity for each outpatient visit was determined using a combination of three basic domains: history, physical examination, and MDM. The lowest complexity score (ranging from 1 to 5) of these three domains was used to determine the overall visit LOS. The history and physical examination sections required extensive documentation to meet higher complexity criteria. For example, all level 4 or 5 visits required the following history documentation: four or more elements of the history of present illness (HPI), 10 or more elements of the review of systems (ROS), and past medical, family, and social histories. A level 4 or 5 musculoskeletal examination required documentation of at least 30 bullet points, including specific minimums in each of the following areas: constitutional, cardiovascular, lymphatic, integumentary, musculoskeletal, and neurologic/psychiatric. These do not usually pertain to most hand surgical complaints. A level 3 visit required less ROS elements (two to nine), only a single past medical, family, or social history documented, and only 12 physical examination bullet points.

It is clear that the recent E/M documentation requirement changes made by the AMA and CMS have had a substantial impact on LOS code selection patterns in our hand and wrist surgery division. With the new emphasis on MDM and added flexibility regarding the history and physical examination documentation, our surgeons have consistently selected higher code complexities consistent with the medical complexity in a very focused, subspecialized field of surgery. Taking all patient encounter types into account, we saw a substantial increase in level 4 visits (CPT E/M code 99204 or 99214) from 7.8% in the 2019 study period to 50.5% in 2021. A corresponding decrease in level 3 visits (CPT E/M code 99203 or 99213) from 84.8% in 2019 to 47.3% in 2021 was noted (Table 2). These trends remained consistent, regardless of insurance type or patient status (new vs. established). Level 2 visits saw a similar decline from 7.2% of all visits in 2019 to less than 2% in 2021. Level 1 and 5 visits remained rare selections at less than 0.5% of all visits.

While CPT coding represents the “common language” for medical procedures and is essential to communication, data collection, and clinical research, this system is also closely tied to reimbursement and valuating healthcare services [5,8,9]. The Relative Value Scale Update Committee (RUC), which is made up of select physician representatives from most medical and surgical specialty societies, plays a major role in determining the value of medical services and procedures. Valuation is based on three primary components: physician work, practice expense, and professional liability insurance (PLI) [8-10]. Each year, the RUC is tasked with updating CPT code valuation recommendations to CMS through a strict methodology. The CMS operates under a rule of budget neutrality, meaning that the expansion in reimbursement for one procedure or service may impact the reimbursement of others [9]. CMS publishes its decisions on any proposed RVU changes and adjusts its annual conversion factor in the Physician Fee Schedule Final Rule each November [2,7,9].

As a result of the increase in LOS coding in our practice, there was a notable increase in mean RVUs per office visit in our hand surgery practice. The most substantial increase was noted in established patient visits, which saw an RVU increase of 65.2% on average (0.96 RVUs in 2019 to 1.59 RVUs in 2021). New patient visits had a mean RVU increase of 43.5% from 2019 to 2021. Insurance type did not portend any major differences in RVU increase, as all four sub-categories had significant increases in mean RVUs from 49.1% to 59.8% (Table 4).

Tassavor et al. compared dermatology resident clinic E/M code level selection patterns between two separate two-month periods before and after the recent changes by the AMA and CMS on January 1, 2021 [11]. After analyzing over 2500 unique patient visits, they reported a similar, but smaller 13% increase in level 4 visits and a 20% decrease in level 2 visits following the recent criteria changes.

Our study has several limitations. There were unprecedented changes in our hand surgery clinic patient flow as a result of the COVID-19 pandemic, requiring a significant amount of telehealth visits [12]. For this reason, we chose to exclude the year 2020 for analysis and instead chose a four-month pre-pandemic timeframe. However, E/M coding principles prior to January 1, 2021, were mostly unchanged for two decades. While it would have been ideal to compare consecutive years, it is unlikely that our results would have differed significantly. Additionally, this study only represents an individual practice’s patient population in the northeastern United States and may or may not apply to other geographies. While the trend of increased coding complexity and RVUs was widespread across all patient visit types and insurance categories, it could be worthwhile to analyze the percentage of approved reimbursement between these groups. The present study did not investigate this. Finally, while all visit level coding was subject to our practice’s standard auditing process, it remains a possibility that billing errors were made.

## Conclusions

It is unknown how uniform these recent coding patterns are among hand surgeons. Individual surgeons and practices may adapt to the regulation changes at different speeds, and LOS selection differences may become even more apparent over time. We suspect that higher complexity code selection since January 1, 2021, will become consistent across the orthopedic subspecialties; however, further investigation in this area is warranted. These findings may have been anticipated by the AMA and CMS following their simplification of documentation guidelines aimed at diminishing the administrative burden on the practicing physician. It remains to be seen what impact these trends have on future reimbursement policies and the healthcare system as a whole. What is clear, however, is that at our institution, since the E/M coding criteria overhaul beginning in 2021, there has been a significant trend toward a higher level of service code selection in hand surgery.
